# Cerebral Pain Processing Following TNFα Inhibitor Treatment in Rheumatoid Arthritis: A Randomized Double‐Blind Placebo‐Controlled fMRI Study

**DOI:** 10.1002/acr2.90069

**Published:** 2026-05-20

**Authors:** Julie Klinke, Peter Fransson, Karin B. Jensen, Reem Altawil, Eva Kosek, Jon Lampa

**Affiliations:** ^1^ Department of Clinical Neuroscience Karolinska Institutet Stockholm Sweden; ^2^ Department of Medicine, Rheumatology Unit, Center for Molecular Medicine (CMM) Karolinska Institutet; ^3^ Department of Gastroenterology, Dermatology and Rheumatology Karolinska University Hospital Stockholm Sweden; ^4^ Department of Surgical Sciences Uppsala University Sweden

## Abstract

**Objective:**

This study investigated the effects of tumor necrosis factor α (TNFα) blockade treatment, adalimumab (ADA), on cerebral pain processing in relevant brain regions in patients with rheumatoid arthritis (RA). Its secondary aim was to identify pretreatment markers in cerebral pain processing that might predict treatment response.

**Methods:**

A total of 25 patients with RA were randomly allocated treatment with 40 mg ADA (n = 14) or placebo (PBO) (n = 11) injected subcutaneously. During functional magnetic resonance imaging (fMRI), patients underwent individually calibrated pressure‐pain stimulation on the most inflamed interphalangeal joint and on an unaffected control site before and after four weeks of treatment.

**Results:**

The ADA but not the PBO group showed significant changes in subjective, clinician‐rated, and objective measures. Both groups exhibited significant activations in pain‐relevant brain regions during the fMRI task. Cerebral activations did not change following treatment nor did they correlate pre‐ or post‐treatment with changes in systemic inflammation (measured as the change in erythrocyte sedimentation rate [ΔESR]). Baseline cerebral activations did not correlate with treatment response in terms of ΔESR nor with pain or fatigue outcomes. However, in an exploratory analysis, five baseline activation clusters in the ADA group, and one in the PBO group, correlated positively with changes in swollen joint count (*P* < 0.001).

**Conclusions:**

Our study did not observe any associations between treatment with TNFα inhibitors and changes in cerebral pain processing, indicating that this treatment may not have a clear central effect.

## INTRODUCTION

Rheumatoid arthritis (RA) is an autoimmune inflammatory pain disease characterized by reduced immune tolerance against self‐proteins and peripheral synovial inflammation that causes erosion of the bones and cartilage of the affected joints.^1^, ^2^ Biologic treatments that aim to inhibit proinflammatory cytokines such as tumor necrosis factor α (TNFα), interleukin (IL)‐1β, and IL‐6 have been associated with decreased RA disease activity.^1^, ^3^, ^4^ However, despite well‐controlled peripheral joint inflammation, some patients continue to report pain. Because pain is one of the most debilitating symptoms of RA,^5^ it is crucial to understand why controlling inflammation does not always provide adequate pain relief.

It has been suggested that central nervous system mechanisms may play a role in RA pain.^6^, ^7^, ^8^, ^9^ There is preliminary evidence that inhibiting TNFα may reduce RA pain partly through central actions. For example, a study by Hess et al^10^ (n = 5) indicated that patients with RA had normalized activity in the somatosensory cortex within 24 hours of TNFα blockade treatment initiation. Rech et al^11^ found that in patients with RA (n = 10) undergoing open‐label treatment, TNFα inhibitor responders could potentially be distinguished from nonresponders based on a higher baseline blood‐oxygen‐level‐dependent (BOLD) activation of the primary somatosensory cortex (S1), dorsolateral prefrontal cortex, and insula during joint compression. In a recent randomized controlled functional magnetic resonance imaging (fMRI) study (n = 139),^12^ patients were stratified based on pain‐related brain volume activation and assigned treatment with TNFα inhibitor or placebo (PBO). The authors found that patients with a high number of brain voxel activation at baseline showed greater treatment response after 12 weeks.

We previously demonstrated that compared to healthy controls, patients with RA exhibited reduced BOLD amplitudes in somatosensory regions, specifically S1, the secondary somatosensory cortex (S2), and insula, when painfully stimulated over their most inflamed finger joint.^13^ In contrast, we did not find such differences between patients with RA and healthy controls in response to painful stimulation at the unaffected thumbnail.

The current study investigates the effects of a four‐week randomized, double‐blind, PBO‐controlled TNFα blockade treatment on pain‐evoked cerebral processing. We applied painful pressure before and following treatment with a TNFα inhibitor, adalimumab (ADA; Humira) or PBO. We hypothesized that TNFα treatment would affect pain‐evoked neural responses in brain regions implicated in pain processing. Furthermore, we hypothesized that the degree of change in neural responses would correlate with an improvement in clinically relevant parameters including levels of systemic inflammation, perceived pain, clinician‐rated assessments of disease activity, and fatigue, because there is evidence of the latter being reduced by ADA treatment.^14^ Our secondary aim was to identify pretreatment markers in cerebral pain processing that might predict the treatment response in patients with RA, as suggested by a previous study.^11^

## PATIENTS AND METHODS

Ethical approval was obtained from the Regional Ethics committee in Stockholm, Sweden (2010/412‐31/1). Clinical trial registration is available on clinicaltrials.gov (NCT01197144). Individual data from this study are not available for sharing because of patient confidentiality and ethical constraints. Aggregated group data are available upon request.

### Patients

Patients with RA were recruited through the rheumatology clinic at Karolinska University Hospital in Stockholm, Sweden. Patients fulfilling the inclusion criteria were asked to participate in a double‐blind, randomized, PBO‐controlled trial investigating the effects of ADA on inflammation and pain in RA. Pretreatment fMRI data from the RA cohort have previously been published in comparison to healthy controls^13^, ^15^ and patients with fibromyalgia.^16^ The current study is the first to report any treatment‐related fMRI data.

**Figure 1:**
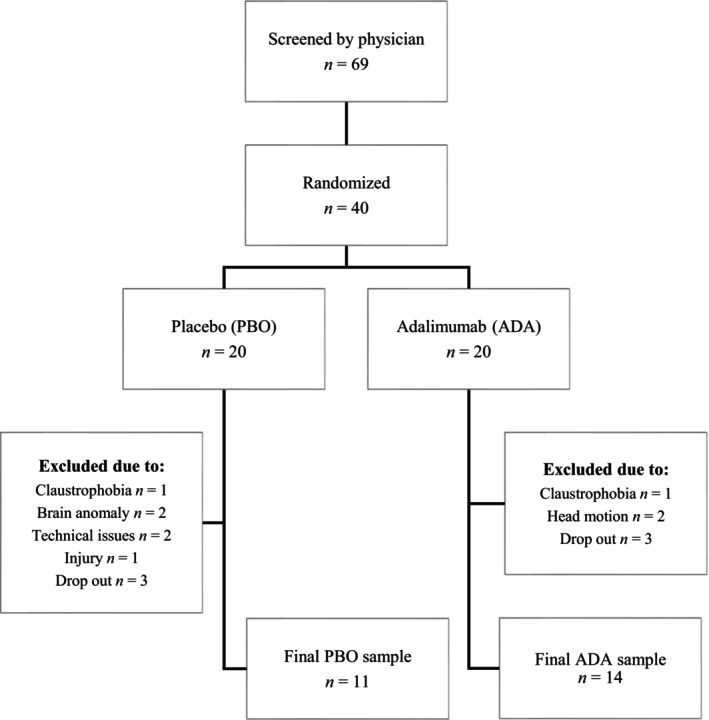
Patient flowchart. Flowchart over patient recruitment, enrollment, randomization, and withdrawal.

Screening of patients with RA was performed by a rheumatologist. Eligible patients fulfilled the American College of Rheumatology 1987 classification criteria for RA,^17^ had clinical indication for use of TNFα inhibitors, were adults (≥18 years), and were approved for magnetic resonance imaging (MRI) examination. Exclusion criteria were left‐handedness, neurologic disease, ongoing treatment with antidepressants, severe cardiovascular disease, fibromyalgia comorbidity, latent tuberculosis, claustrophobia, or other motives based on the judgment of the responsible physician (Figure 1). Informed consent was obtained from all participants.

Of the 69 screened patients with RA, 40 were enrolled in the study and randomized to either ADA (n = 20) or PBO (n = 20) in a double‐blind manner. Following exclusions due to claustrophobia, brain anomalies, excessive head motion, and drop‐outs, the final sample included ADA: n = 14 (12 women; mean age = 55, SD = 13) and PBO: n = 11 (9 women; mean age = 54, SD = 16) (Figure 1).

### Procedure

The study protocol consisted of six visits separated into two blocks, pre‐ and post‐treatment, with three visits taking place during each block (see Supplementary Figure 1). During visits 1 and 4, all participants met with a rheumatologist and a research nurse to fill in questionnaires, undergo a physical examination of their disease activity, and have their blood samples taken to measure their erythrocyte sedimentation rate (ESR) and C‐reactive protein (CRP). All other visits were conducted at the MRI Research Center at Karolinska Institutet and the Karolinska University Hospital, Stockholm, Sweden. During the second visit of each block, patients with RA’ subjective pressure‐pain intensities were individually calibrated. During the third visit of each block, the patients with RA underwent fMRI scanning. The fMRI scanning visits (third of each block) were scheduled for the day after the calibration visits (second of each block). After the pretreatment block, patients returned to the rheumatology clinic to start the randomized treatment with either ADA (40 mg subcutaneously every other week) or PBO injections. Each patient received a total of three injections before the second, post‐treatment block: one injection at baseline, one two weeks after baseline, and one four weeks after baseline.

#### Clinical outcomes

RA disease activity was measured with Disease Activity Score based on 28 joints (DAS28), which combines clinician ratings of tender joint count (TJC) and swollen joint count (SJC), laboratory measures of CRP or ESR, and patient‐rated global health.^18^

Pain and fatigue were measured with visual analog scale (VAS), a one‐dimensional self‐assessment instrument consisting of a 100‐mm scale anchored by 0 (“no pain” or “no fatigue”) to 100 (“worst imaginable” pain or fatigue).^19^ VAS ratings for weekly pain on average, on average at night, at mildest, at worst during movement, and at rest, as well as for severity of weekly fatigue, were collected at the first visits pre‐ and post‐treatment. Current pain rating was collected on the days of the fMRI scan. Pain variability was calculated by subtracting patients’ VAS ratings of mildest pain from worst pain.

Fatigue was also measured with the self‐report instrument multidimensional assessment of fatigue, which measures fatigue in patients with RA through four dimensions: degree or severity, distress, timing, and impact on daily activities. A higher score indicates a higher level of fatigue.^20^

Health‐related quality of life (HRQOL) was measured with two questionnaires: EuroQol 5 Dimension (EQ‐5D) and the 36‐Item Short Form Survey (SF‐36). EQ‐5D measures HRQOL via five dimensions: mobility, self‐care, usual activities, pain or discomfort, and anxiety or depression.^21^ SF‐36 measures quality of life on eight domains: physical functioning, role limitations due to physical health problems, emotional well‐being, social functioning, role limitations due to personal or emotional problems, bodily pain, vitality (energy or fatigue), and general health perceptions. Low scores indicate reduced quality of life.^22^

Sleep quality was assessed with Pittsburgh Sleep Quality Index (PSQI), a self‐report instrument measuring seven components of global sleep quality: subjective sleep quality, sleep latency, sleep duration, habitual sleep efficiency, sleep disturbances, use of sleeping medication, and daytime dysfunction. A higher score indicates worse sleep quality.^23^

#### Subjective calibration of pressure‐pain stimuli

Pressure sensitivity was individually calibrated to determine the amount of pressure (kPa) that corresponded to a subjective rating of 50/100 mm VAS for each patient before treatment (visit 2) and following treatment (visit 5). This was done to ensure that the analyses were optimized for analyzing cerebral pain processing and avoid variance attributed to interindividual differences in pain sensitivity.

All pressure stimuli were delivered using an automated, computer‐controlled plastic cylinder with a 1 cm^2^ hard rubber probe. The probe was placed at the most inflamed proximal interphalangeal (PIP) joint on the left hand (assessed by a rheumatologist) at baseline (PIP2 n = 21; PIP3 n = 4) as well as on the nonaffected left thumbnail (control site). The same joint was used at follow‐up. First, patients received one ascending series of pressure stimuli, with increasing steps of 50 kPa, to determine the pressure‐pain threshold (PPT, first VAS > 0 mm) and stimulation maximum (SM, first VAS > 60 mm) at each site. The ascending series was followed by three randomized series, in which five different pressure intensities were calculated and delivered within the range of each patient's PPT and SM pressures. In all series, each pressure stimulus was applied for 2.5 seconds with 30‐second intervals. Following each stimulus, patients were asked to rate their perceived pain intensity using the VAS. Finally, polynomial regression was used to fit the 15 subjective pain ratings from the randomized series to determine each individual representation of 50 mm VAS (P50) for the joint and the thumbnail, respectively. For further information, see Sandström et al.^13^

### fMRI acquisition

MRI images were acquired using a 3T General Electric 750 MRI scanner installed at the MR Research Center, Karolinska Institutet, Karolinska University Hospital, Stockholm, and fitted with a 32‐channel head coil. Patients were scanned before treatment initiation (visit 3) as well as four weeks following treatment initiation (visit 6). Anatomic MRI scans were acquired in both groups with a high‐resolution BRAVO 3D T1‐weighted image sequence (1 × 1 × 1 mm^3^ voxel size). Anatomic T2‐weighted scans were collected so a neuroradiologist could screen participants for clinical abnormalities. Functional images covering the whole brain were acquired following the anatomic scans using a T2*‐weighted single‐shot gradient echo planar imaging sequence, interleaved axial slice acquisition, number of slices = 56, slice thickness = 3 mm, flip angle = 90°, 96 × 96 matrix size, field of view = 288 × 288 mm, time to repetition or echo time = 3,000/30 ms.

Patients underwent fMRI scanning during the pressure‐pain stimulation paradigm, in which they were stimulated with their individually calibrated painful pressure (P50) and a fixed nonpainful pressure (50 kPa) over their most inflamed finger joint and their nonaffected left thumbnail. The fixed nonpainful pressure was included for the purpose of having an active baseline for comparison in fMRI analysis to refine pain‐specific neural activations.

Participants underwent four fMRI runs of pressure stimulations at each fMRI visit. Two runs stimulated the joint and two runs stimulated the thumb. Each run consisted of 30 pressure stimuli (15 painful and 15 nonpainful). All pressures were delivered for 2.5 seconds each in a pseudorandomized order jittered over time. Mean interval between stimuli onsets was 15 seconds (range 10–20 seconds). Each fMRI run had a total duration of 8 minutes and 15 seconds.

At the end of each scanning session, resting‐state data were collected. Baseline resting‐state data have been reported elsewhere.^15^

### Patient and public involvement

The study did not involve patient or public involvement in its design, analysis, or interpretation.

### Statistics

#### Clinical characteristics

Clinical characteristics and behavioral data were analyzed using IBM SPSS Statistics (version 29.0.1.0). Because the non‐Gaussian distribution of clinical characteristics within‐group (pre/posttreatment) and between‐group (ADA/PBO) were determined using the Shapiro–Wilk test of normality, the clinical data were analyzed using nonparametric statistics (Table 1; for additional information, see Supplementary Table 1). Differences between the groups’ demographic variables and clinical characteristics were calculated using an independent‐samples Mann–Whitney U test. Within‐group differences in clinical characteristics were calculated using a (paired) Wilcoxon Signed Rank's test.

**Table 1:** Clinical characteristics*

Clinical characteristics	ADA, n = 14			PBO, n = 11			ADA vs PBO	
	Pre	Post	*P*	Pre	Post	*P*	Pre	Post
Age, mean (SD), y	55 (13)	–	–	56 (15)	–	–	0.809	–
Gender (female/male), n	12/2	–	–	8/3	–	–	–	–
Body mass index, median (IQR)	24 (22–29)	–	–	26 (21–28)	–	–	0.767	–
Disease duration, median (IQR), y	2 (1–4)	–	–	4 (1–8)	–	–	0.373	–
P50 joint, median (IQR)	497 (303–850)	563 (331–850)	0.722	500 (390–736)	646 (500–850)	0.314	0.936	0.647
P50 thumb, median (IQR)	460 (359–601)	538 (392–850)	0.345	555 (371–712)	683 (394–774)	1.00	0.572	0.979
VAS pain at scan, median (IQR)	49 (30–66)	10 (3–32)	0.004^a^	31 (22–53)	35 (14–44)	0.540	0.344	0.202
Pain last week, median (IQR)								
Mildest	16 (7–42)	2 (0–7)	0.023^a^	9 (3–40)	9 (2–29)	0.813	0.727	0.085
Average	58 (29–70)	15 (6–52)	0.006^a^	32 (27–57)	27 (22–53)	0.182	0.344	0.244
Average at night	65 (25–83)	37 (3–50)	0.225	26 (8.5–56)	26 (23–49)	0.180	0.280	0.841
Worst during movement	54 (33–81)	24 (6–57)	0.019^a^	52 (40–75)	55 (23–65)	0.139	0.893	0.291
Rest	61 (29–52)	17 (2–38)	0.012^a^	44 (23–79)	35 (20–48)	0.444	0.373	0.222
Pain variability, median (IQR)	23 (20–41)	21 (5–49)	0.530	29 (24–47)	23 (10–45)	0.450	0.222	0.809
EQ‐5D index, median (IQR)	0.707 (0.088–0.796)	0.725 (0.656–0.796)	0.109	0.727 (0.604–0.796)	00.725 (0.568–0.796)	0.498	0.403	1.00
EQ‐5D VAS, median (IQR), mm	74 (44–83)	90 (79–91)	0.017^a^	66 (42–81)	70 (50–90)	0.635	0.809	0.095
Fatigue VAS, median (IQR)	62 (5–80)	47 (3–66)	0.450	33 (7–67)	22 (5–62)	0.358	0.531	0.531
MAF, median (IQR)	32 (9.8–38)	26 (14–31)	0.173	26 (12–32)	25 (8–31)	0.878	0.317	0.977
PSQI total, median (IQR)	7 (6–12)	6.5 (5–11)	0.107	7 (4–8)	8 (3–8)	0.223	0.291	0.373
SF36‐PCS, median (IQR)	37 (29–44)	42 (36–48)	0.016^a^	38 (30–41)	38 (30–47)	0.374	0.936	0.403
SF36‐MCS, median (IQR)	43 (29–56)	49 (44–56)	0.140	47 (39–56)	48 (39–58)	0.374	0.687	0.767
DAS28 ESR, median (IQR)	5 (4–6)	4 (3–5)	0.002^a^	5 (4–5)	4 (4–5)	0.075	0.317	0.434
DAS28 CRP, median (IQR)	5 (4–5)	3 (3–4)	0.002^a^	4 (4–5)	4 (3–5)	0.505	0.434	0.183
SJC, median (IQR)	6 (4–7)	3.5 (1–5)	0.005^a^	5 (3–7)	3 (2–6)	0.279	0.434	0.536
TJC, median (IQR)	9 (5–16)	4 (1–10)	0.041^a^	6 (5–11)	7 (4–10)	0.122	0.727	0.687
ESR, median (IQR)	34 (19–46)	18 (12–36)	0.032^a^	16 (11–32)	15 (11–27)	0.373	0.222	0.851
CRP, median (IQR)	5 (3–26)	2 (1–6)	0.071	6 (1–10)	5 (1–25)	0.293	0.467	0.373

* CRP, C‐reactive protein; DAS28, Disease Activity Score based on 28 joints; EQ‐5D, EuroQol 5 Dimension; ESR, erythrocyte sedimentation rate; IQR, interquartile range; MAF, Multidimensional Assessment of Fatigue; MCS, mental health component score; P50, pressure corresponding to 50/100 pain rating; PCS, physical health component score; PSQI, Pittsburgh Sleep Quality Index; SF‐36, 36‐Item Short Form Survey; SJC, swollen joint count; TJC, tender joint count; VAS, visual analog scale.

^a^ Significance at *P* < 0.05.

#### fMRI data analysis

Imaging data analyses were performed using the Statistical Parametric Mapping 12 (SPM12) software^24^ (http://www.fil.ion.ucl.ac.uk/spm/software/spm12/) running in MATLAB (version R2022a). MarsBaR version 0.45 was used for beta value extraction.^25^ The restricted search volume, region of interest (ROI), for the present study was created based on structural regions from the automated anatomic labeling (AAL) atlas within the WFU PickAtlas toolbox in SPM12. The ROI was based on our previous report of significantly different activations in patients with RA as compared to healthy controls in response to painful stimulation.^13^ Because our previous study included the same patients with RA as the present study (although only at baseline), we defined our a priori brain regions independently using the AAL atlas to avoid circularity. Our ROI included the bilateral pre and postcentral gyri, supramarginal gyri, insulae, mid cingula, and paracentral lobules. Outputs of fMRI analyses were visualized using MRIcroGL (version 1.2) and MRIcron (version 1).

For the main outcomes, fMRI group analyses were run on the contrast [joint pain] – [joint sensory] corrected for multiple comparisons using a family‐wise error (FWE) corrected *P* value of <0.05. Age was added as a covariate of no interest in within‐group analyses but not in between‐group analyses as the ADA and PBO groups were age‐matched. Exploratory analyses (baseline cerebral activation × DAS28, subjective pain variability, fatigue, or clinician‐rated TJC, SJC, and changes in SJC [ΔSJC]) were conducted using a more liberal statistical threshold of *P* < 0.001, uncorrected for multiple comparisons.

##### Neuroimaging data preprocessing

Scans were manually reoriented to the anterior or posterior commissure (AC or PC) line to facilitate the coregistration and spatial normalization process. Functional images were spatially realigned using a six‐parameter affine transformation and registered to the mean. Individual structural images were coregistered with functional images. Coregistered images were normalized to 2‐mm Montreal Neurological Institute space and spatially smoothed using a 6‐mm full‐width–half‐maximum Gaussian kernel. Head motion from one volume to the next (translational and rotational head movement) was assessed using the framewise displacement (FD) parameter. The degree of rotational displacements was converted from degrees to millimeters by calculating displacement on a sphere with a 50‐mm radius. Patients were excluded from data analysis if the FD parameter exceeded 0.5 in more than 15% of the images in each run during pre or posttreatment scans (leading to the exclusion of two patients receiving ADA) (see Figure 1).

##### Neuroimaging data analysis

The first level general linear model included our experimental regressors of interest convolved with the canonical hemodynamic response function, including separate estimations for painful and nonpainful joint pressures. Six motion parameters were added as regressors of no interest.

At the second level, a 2 × 2 full factorial analysis of variance (ADA/PBO[group] × pre/post treatment[time]) was used to examine whether brain activation in response to pain changed as an effect of treatment. In this analysis, time was set as a repeated‐measures variable to exploit the full power benefit of within‐subject scans.

Rech et al found that patients with RA with higher baseline cerebral activations may respond better to TNFα treatment.^11^ Their analysis was based on dichotomized groups (responders or nonresponders) and did not represent the full range of responses. Because dichotomizing linear values has been shown to reduce statistical power of up to one‐third,^26^ the present study instead conducted the analysis with change in systemic inflammation (change in erythrocyte sedimentation rate [ΔESR]) as a continuous variable. These analyses were first run on our a priori ROI and then exploratively on the whole brain. In addition to the objective ΔESR measurement, an a priori regression analysis with VAS fatigue as the regressor was performed on the pain‐evoked brain activation data at baseline, separately for the ADA and PBO groups. Finally, four exploratory regression analyses were performed separately for the ADA and PBO groups on their baseline activation data, with pain variability, SJC, ΔSJC, and TJC as regressors.

## RESULTS

### Effects of treatment

#### Clinical characteristics of patients pre and posttreatment

There were no statistically significant differences between patients with RA randomized to ADA or PBO treatment at baseline for demographic data nor for subjective, objective, or clinician‐rated measures of pain, disability, and quality of life (*P* < 0.05). Within‐ and between‐group scores and *P* values for clinical characteristics pre and posttreatment, including demographic, subjective, objective, and clinician‐rated measurements, are summarized in Table 1. Following treatment, only the ADA‐treated group showed significant changes in both subjective, clinician‐rated, and objective measures in the form of reduced pain over the past week, improved physical quality of life, and reduced disease activity scores and TJC and SJC, *P* < 0.05. However, there were no significant between‐group differences at follow‐up in a direct comparison. There were no significant within‐ or between‐group differences in P50 before or after treatment (Table 1; Supplementary Figure 2).

#### Adverse events

The presence of adverse events (AEs) in all patients who started ADA treatment, regardless of whether they were included in the fMRI part of the study (N = 36; ADA n = 18; PBO n = 18), is presented in Supplementary Table 2. Note that AE reports had a follow‐up period of 12 weeks as opposed to 4 weeks for all other outcomes.

In summary, 33 AEs were reported for ADA and 14 AEs for the PBO group. The AEs included four patients (two ADA and two PBO) with serious infections. Two severe AEs were reported: one patient developed breast cancer (PBO group, excluded after week 4), and one patient underwent physical trauma with head and arm fracture (ADA group, excluded from fMRI assessment week 4). More than one AE may be reported by the same patient.

### fMRI

#### Pain‐related functional brain activation as an effect of group and time

An F‐test showed significant pain‐related brain activation in response to painful stimulation of the joint regardless of group and time (Supplementary Figure 3). Significant clusters are reported in Table 2 and include the right postcentral gyrus and the right insula.

**Table 2:** F‐contrast: [joint pain] – [joint sensory], ROI analysis*

Cluster location	MNI peak coordinates			Cluster size	*F*‐value	*z*‐score	FWE‐corrected *P* value
	x	y	z				
R Postcentral	52	−20	52	283	53.19	5.80	<0.001
*R Precentral*	40	−18	44		52.44	5.77	
R Insula	40	−14	16	4	35.50	4.97	0.013

* Table shows 2 × 2 analysis of variance: PBO versus ADA [group] × PainPRE versus PainPOST (time). ADA, adalimumab; FWE, family‐wise error; MNI, Montreal Neurological Institute; PBO, placebo; ROI, region of interest.

The full factorial design demonstrated no significant effect of time or group or interaction. Thus, pain‐evoked cerebral processing did not change across groups before and after treatment. Results of brain activation for each group and time point that did not survive FWE correction are illustrated for completeness in Supplementary Figure 3.

#### Within‐group pain‐related brain activation and change in systemic inflammation

**Figure 2:**
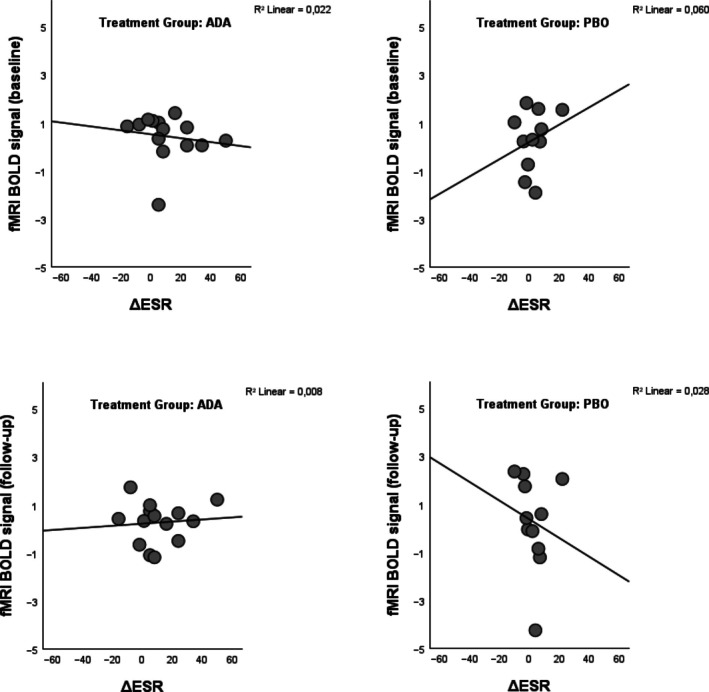
Correlation between pain‐evoked brain activity and change in systemic inflammation. The *x*‐axis represents the change in ESR from baseline to follow‐up. The *y*‐axis represents extracted fMRI BOLD signal from the pain ROI at baseline (top row) and follow‐up (bottom row). ADA, adalimumab; BOLD, blood‐oxygen‐level‐dependent; ΔESR, change in erythrocyte sedimentation rate; fMRI, functional magnetic resonance imaging; PBO, placebo.

A regression analysis did not find any baseline brain activation that correlated with treatment‐induced changes in systemic inflammation (ΔESR) within either group, neither in the ROIs nor whole brain. For transparency, extracted beta values from baseline fMRI and ΔESR values are shown in Figure 2.

#### Within‐group pain‐related brain activation and disease‐relevant measurements at follow‐up

**Figure 3:**
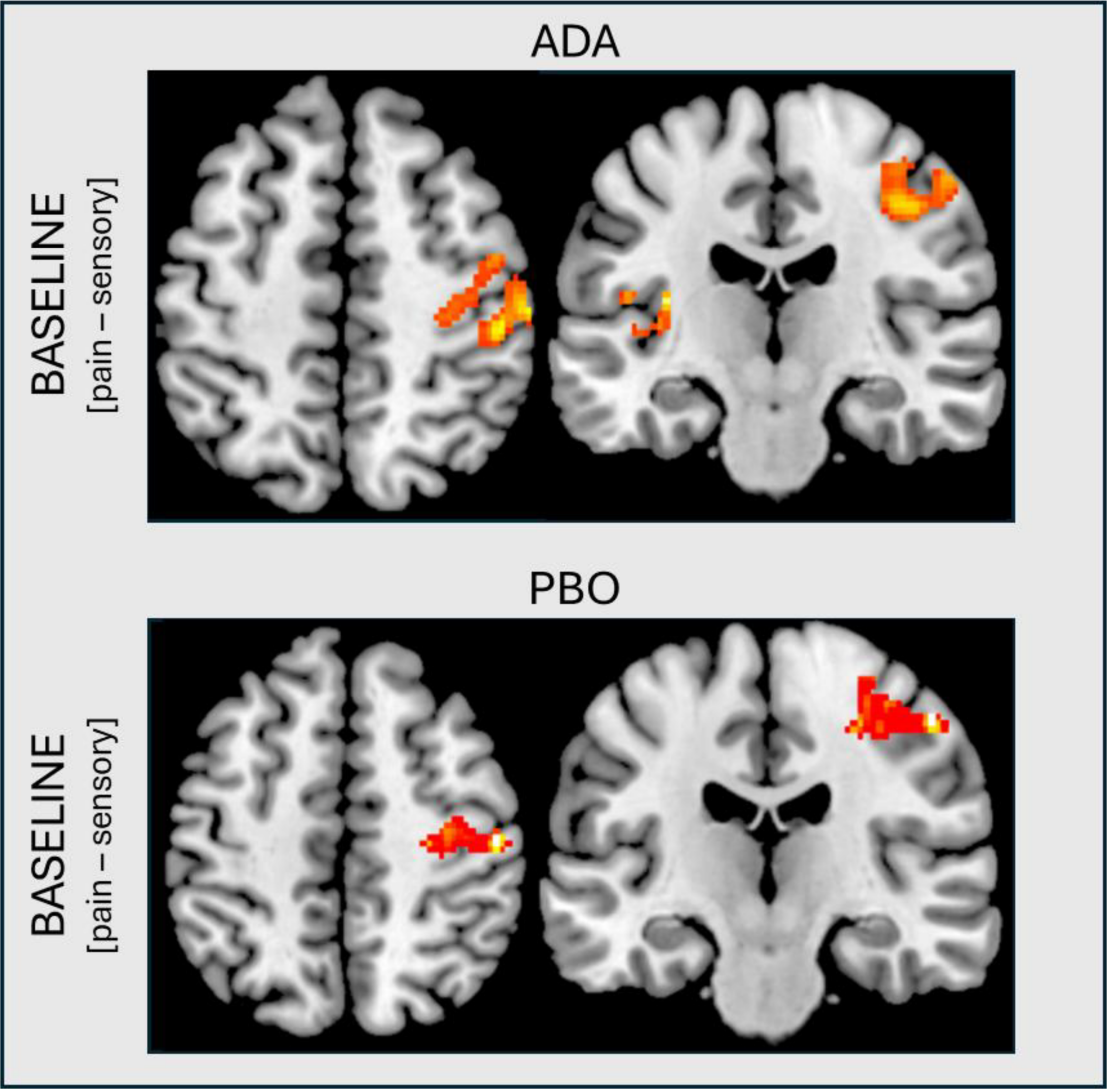
Increased activation clusters positively correlated with changes in SJC. Figure illustrates locations of pain‐evoked brain activations that were positively correlated with changes in swollen joint counts (baseline minus follow‐up). All active clusters are represented at *P* < 0.001, uncorrected, >100 contiguous voxels, whole‐brain level. Heat maps visualize activation peaks and do not represent t‐scores or z‐scores (for this, see Table 3). ADA, adalimumab; PBO, placebo; SJC, swollen joint count.

Five baseline activation clusters in the ADA group, and one in the PBO group, correlated positively with ΔSJC (*P* < 0.001), uncorrected for multiple comparisons. Their anatomic locations are illustrated in Figure 3. Exact coordinates of activation peaks, cluster sizes, t‐scores, and z‐scores are shown in Table 3.

**Table 3:** Regression analysis: baseline increased activation × ΔSJC*

Cluster location	MNI peak coordinates			Cluster size	*T*‐value	*z*‐score	FWE‐corrected *P* value
	x	y	z				
ADA: whole brain							
L rolandic operculum	−54	−2	6	627	12.44	5.37	<0.001
L insula	−34	−18	14		7.47	4.37	
L cerebellum	−24	−58	−36	279	8.58	4.65	0.001
L cerebellar vermis	−4	−62	−16	199	8.45	4.62	
R precentral cortex	52	−12	44	667	6.97	4.23	<0.001
R postcentral cortex	46	−28	54		6.56	4.10	
R temporal lobe	56	0	4	306	6.79	4.17	<0.001
R frontal inferior operculum	48	18	0		6.58	4.11	
R temporal pole	54	8	−4		5.98	3.91	
ADA: ROI							
R precentral cortex	52	−12	44	631	6.97	4.23	<0.001
R postcentral cortex	46	−28	54		6.56	4.10	
PBO: whole brain							
R postcentral cortex	50	−20	50	289	27.31	5.91	<0.001
R precentral cortex	36	−22	56		7.99	4.08	

* T‐contrast, whole brain, exploratory post‐hoc, not corrected for multiple comparisons. Findings were identical for whole‐brain and ROI analyses (inclusive mask) in the PBO group. ADA, adalimumab; FWE, family‐wise error; MNI, Montreal Neurological Institute; PBO, placebo; ROI, region of interest.

No baseline pain‐evoked cerebral activations correlated with follow‐up measures of DAS28, subjective pain variability, fatigue, or clinician‐rated TJC and SJC, in our ROIs or at a whole‐brain level (*P* < 0.001), uncorrected for multiple comparison. For completeness, activation‐related beta values and nonsignificant outcome measures of interest have been illustrated in Supplementary Figure 4.

## DISCUSSION

### General

To our knowledge, this is the first trial comparing effects of a TNFα inhibitor in RA versus PBO on pain‐evoked cerebral activations in pain‐relevant brain regions. As expected, ADA treatment was associated with suppression of inflammation and disease activity and with clinical improvement. However, TNFα inhibitors were not associated with changes in pain‐evoked cerebral activations in this cohort. The brain activations of the two groups largely overlapped from pre‐ to post‐treatment.

In an exploratory, post hoc analysis, our study found preliminary indications that pain‐evoked brain activations at baseline serve as predictors of clinician‐rated symptom improvements in the form of ΔSJC. Activations in multiple brain areas at baseline correlated with ΔSJC, with higher activations at baseline correlating with stronger improvements at follow‐up in both groups.

### fMRI

The present study did not observe any clear associations between ADA‐induced immune suppression and pain‐evoked cerebral processing. Although we have previously found that patients with RA exhibit differential pain‐evoked brain activations compared to healthy controls,^13^ treatment‐induced suppression of inflammation was not coupled with reduced pressure‐pain sensitivity or changes in cerebral pain activity. This is in contrast to previous studies, which have shown that systemic inflammation can modulate pain thresholds in healthy participants.^27^

The present study shows how persistent pain and inflammation in RA does not have a linear relationship, possibly due to adaptive processes and chronification. Here, RA‐related inflammation and persistent pain did not appear to be directly coupled to cerebral pain processing as there was no change in pain‐evoked brain activity after successful suppression of systemic inflammation. However, baseline pain‐evoked cerebral processing correlated with changes in SJC after treatment with TNFα.

Our findings did not follow previous reports of treatment‐related changes to cerebral pain processing. Previous findings have suggested that TNFα inhibitors may be able to alter cerebral pain processing and that baseline cerebral processing could help determine which patients could benefit from TNFα inhibitor treatment.^10^, ^11^ A recent large‐sample (n = 139) multicenter study investigating another TNF‐blocking agent, certolizumab pegol, found that when stratifying patients by pain‐evoked activation volume at baseline, those in the “high volume activation” group showed greater responses at 12‐week follow‐up than those in the “low volume activation” or PBO group.^12^

There are several possible reasons why we did not observe similar patterns. First, the studies by Hess et al^10^ and Rech et al^11^ had small sample sizes and could not apply conservative statistical approaches, increasing the risk of false positives. Second, Rech et al divided participants into responders versus nonresponders by dichotomizing a continuous outcome variable, thereby limiting the study's ability to detect statistical relationships between neural and clinical responses.^26^ Third, both lacked a PBO control cohort. Finally, unlike any existing reports, the present study included an active baseline by interleaving painful and nonpainful pressure stimulation. This may have affected comparability.

The recent multicenter study by Hess et al^12^ had a much larger sample size than our study, implying increased sensitivity to detect differences in cerebral activation patterns between cohorts. Regrettably, due to participant drop‐out, our study was likely underpowered to replicate any differences between cohorts based on baseline measures of pain‐evoked brain activation. However, several methodologic differences between Hess et al's study and this study deserve mention. First, Hess et al's model was designed to separate groups into high versus low activation volume at baseline, not to distinguish treatment responders from nonresponders.^28^ Second, Hess et al opted not to conduct any ROI analyses but rather looked at changes in BOLD activation volume at a whole‐brain level. This indicates that the authors did not investigate peak activation strength or localization, but rather, stratified participants based on a predefined number of voxels regardless of whether voxels were active in pain‐relevant regions. In contrast, the present study, although smaller in sample size, explicitly investigated differences and changes of activation in regions that have been previously validated as relevant for nociceptive pain processing and chronification.

### Clinical

The treatment with TNF‐blocking agents results in a fast and efficient decrease of systemic inflammation and disease activity in patients with RA, as well as reducing RA‐related fatigue.^14^, ^29^ In this study, as expected, ADA treatment significantly reduced systemic inflammation (ESR) and number of swollen and tender joints, while significantly improving disease activity, pain, and quality of life. However, there was no improvement in fatigue scores. Exploratory analyses did not find any predictive relationships between baseline cerebral activation and changes in all but one clinical outcome (ΔSJC).

### Limitations

The lack of significant group differences in any of the investigated clinical variables following treatment may be limited by the short, four‐week follow‐up period. Although an improvement in disease activity and subjective scales including pain would be expected after 4 weeks, 12 weeks are required for full treatment effectiveness. Thus, it is possible that potential changes to cerebral pain processing would emerge over longer treatment durations. This is what may have happened in the recent multicenter study,^12^ which found differences between baseline and at 12‐week follow‐up. However, for ethical reasons it was difficult to motivate withholding effective treatments in the PBO group. Also, because the background studies had shorter durations,^10^, ^11^ this limitation is not necessarily the cause of different findings.

Although the present study comprises one of the largest fMRI assessments of treatment‐related changes in pain‐evoked cerebral processing in patients with RA to date, the sample size was unfortunately limited. Therefore, the present study was underpowered to detect smaller differences in brain activation. Finally, an impact of steroid treatment cannot be excluded; however, any effects should be limited because no patient received doses higher than 10 mg/day.

### Conclusion

Our findings did not indicate that cerebral pain processing at baseline in RA will be a clinically useful measure for predicting individual short‐term treatment responses to TNFα inhibitors.

## AUTHOR CONTRIBUTIONS

All authors contributed to at least one of the following manuscript preparation roles: conceptualization AND/OR methodology, software, investigation, formal analysis, data curation, visualization, and validation AND drafting or reviewing/editing the final draft. As corresponding author, Dr Klinke confirms that all authors have provided the final approval of the version to be published and takes responsibility for the affirmations regarding article submission (eg, not under consideration by another journal), the integrity of the data presented, and the statements regarding compliance with institutional review board/Declaration of Helsinki requirements.

## Supporting information


**Disclosure Form**:

**Data S1.** Supporting Information.
