# Supplementary material for: Cerebral Pain Processing Following TNFα Inhibitor Treatment in Rheumatoid Arthritis: A Randomized Double‐Blind Placebo‐Controlled fMRI Study
Source: ACR Open Rheumatol. 2026 May 20;8(5):e90069. doi: 10.1002/acr2.90069 (PMC13239608; doi:10.1002/acr2.90069)

| **Suppl Table 1** | | | | | |
| --- | --- | --- | --- | --- | --- |
| **Clinical characteristics** | | | | | |
|  | **ADA n = 14** | | **PBO n = 11** | | |
|  | **pre** | **post** | | **pre** | **post** |
| *RF positive (yes/no)* | (11/3) | - | | (10/1) | - |
| *ACPA positive (yes/no)* | (11/3) | - | | (10/1) | - |
| *sDMARD MTX monotherapy (n)* | 10 | 10 | | 7 | 7 |
| *sDMARD other monotherapy # (n)* | 2 | 2 | | 0 | 0 |
| *sDMARD combo # # (n)* | 1 | 1 | | 3 | 3 |
| *no DMARD (n)* | 1 | 1 | | 1 | 1 |
| *NSAID user (regular/if needed/no)* | 5/5/4 | 3/7/4 | | 1/4/6 | 1/4/6 |
| *Analgesics (regular/if needed/no)* | 1/7/6 | 1/7/6 | | 1/6/4 | 1/5/5 |
| *RF = rheumatoid factor; ACPA = anti-citrullinated protein antibodies; sDMARD = synthetic disease-modifying antirheumatic drug; MTX = methotrexate; NSAID = nonsteroid anti-inflammatory drug.* | | | | | |

| **Suppl Table 2** | | |
| --- | --- | --- |
| **Adverse events** | | |
|  | **ADA n=18** | **PBO n=18** |
|  |  |  |
| **Serious Adverse Events** | 1 | 1 |
|  |  |  |
| **Infectious adverse events** |  |  |
| **URI** | 8 | 4 |
| **LRI** | 1 |  |
| **Other** | 1 Oral herpes infection | 1 Gastroenteritis |
|  |  |  |
| **Serious infections** | 1 pt with UVI 4 times, resolved after ab  1 pt with wound infection, ab treated, resolved | 1 pt with Pneumonia, ab treated x 2, resolved  1 pt with skin infection, ab treated, resolved |
|  |  |  |
| **Malignancies** | 0 | 1 pt with breast cancer, see SAE |
|  |  |  |
| **Elevated liver enzymes** | 2 | 2 |
|  |  |  |
| **Skin** |  |  |
| **Injection site reactions** | 4 | 0 |
| **Other** | 1 pt with worsening of psoriasis leading to discontinued treatment  1 pt with wound, not infected |  |
|  |  |  |
| **Other AEs** | 4 Headache  3 Dizziness  1 Depression  1 Atrial fibrillation  1 Migraine  1 Fatigue  1 Nausea, abdominal pain | 1 Headache  1 Hypertension  1 vomiting once  1 arthritis flare |
|  |  |  |
| **Patients reporting no AE** | 1 | 6 |
| *A summary of AEs of all patients who started treatment with the study products (N=36). AEs recorded during the whole study are displayed in table. Note that one patient may have had several AEs.* | | |

**
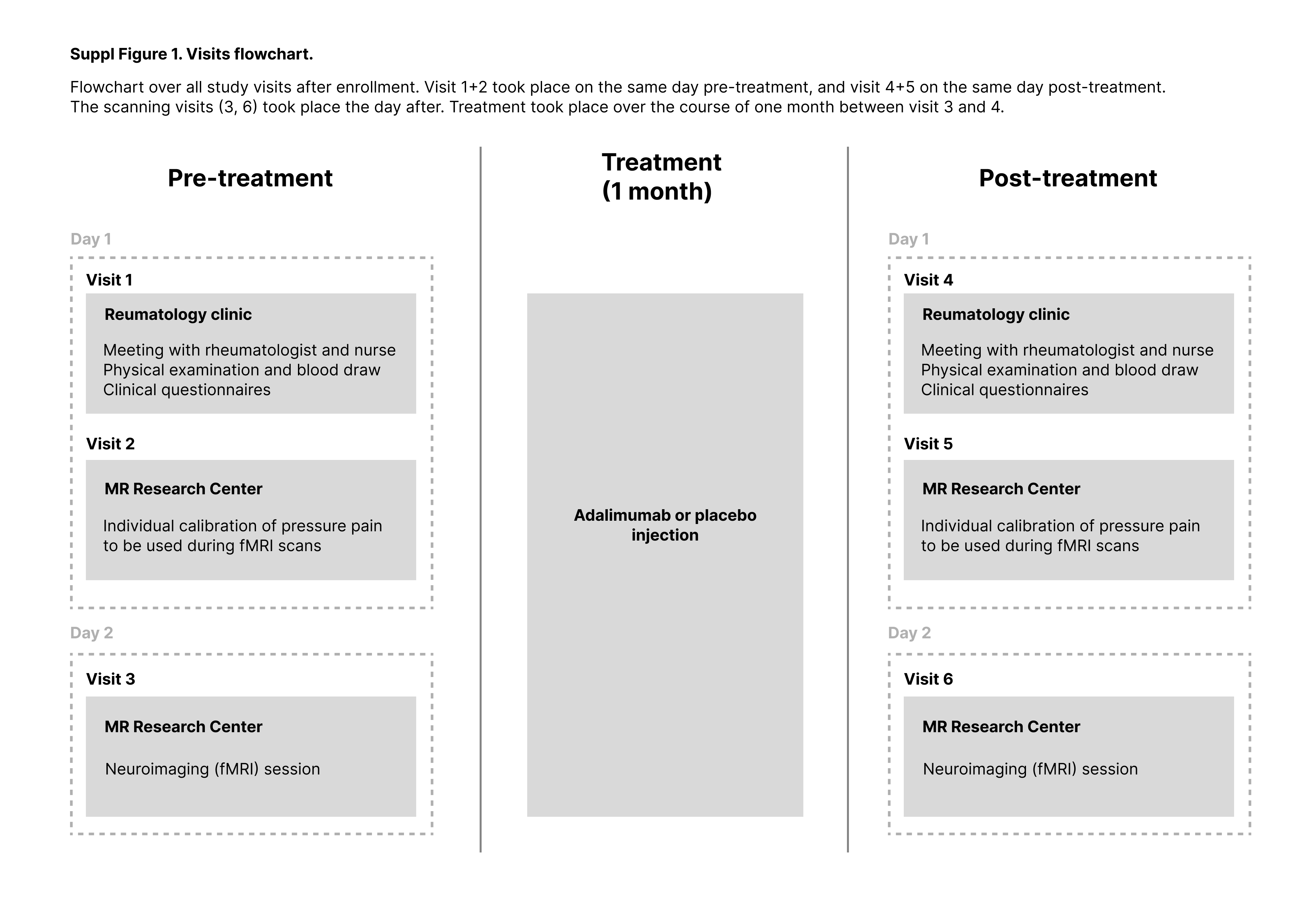
**

**Suppl Figure 2. Thresholds for painful stimulation at the thumb and joint site for ADA vs PBO group.**

**
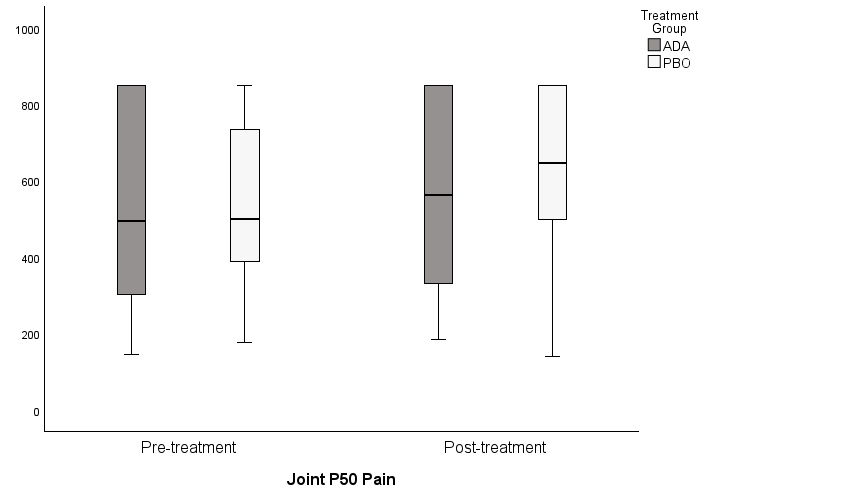
**

kPa

**
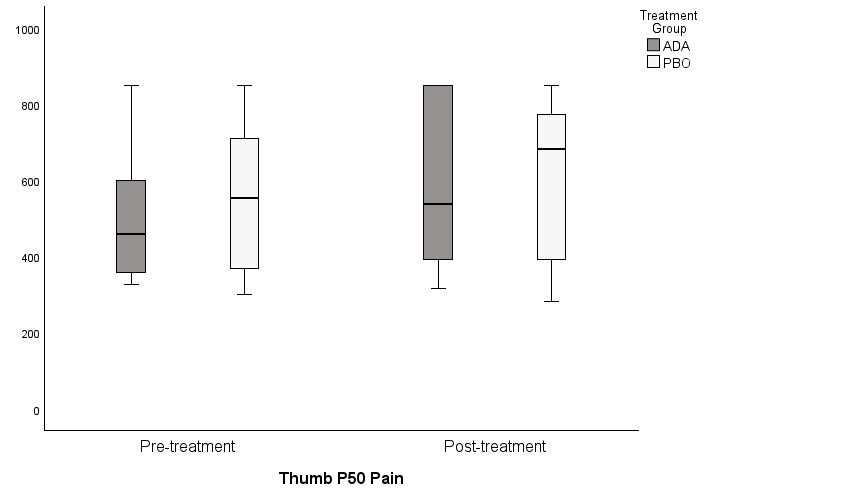
**

kPa

**Suppl Figure 3. Baseline vs follow-up pain-evoked cerebral activations for ADA and PBO.**

**
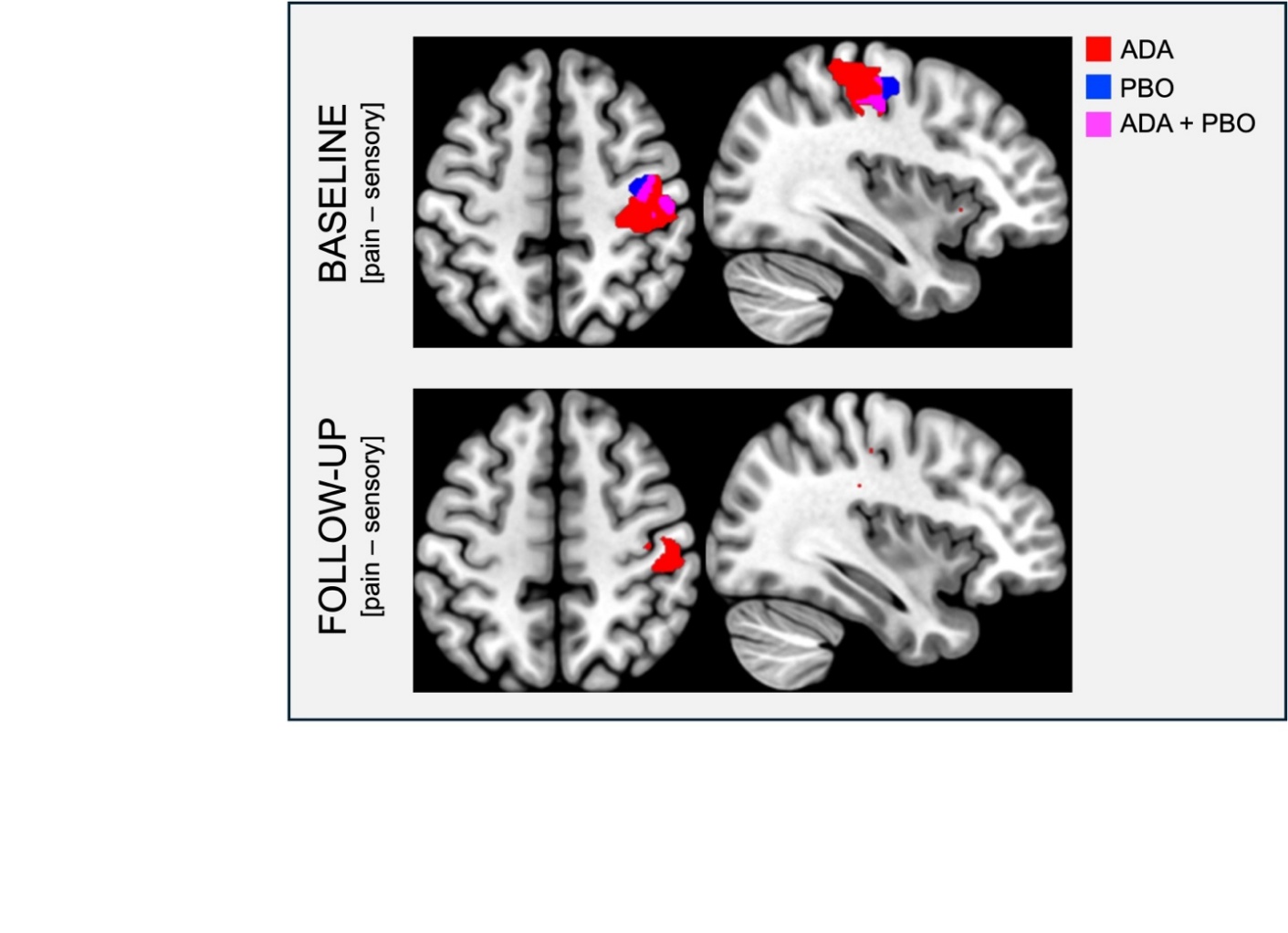
**ADA = red; PBO = blue; overlap = purple. Figure illustrates binary spatial maps of the pain-evoked brain activations. All active clusters are represented at p < .001, uncorrected, >30 contiguous voxels, with an a priori mask applied. No voxels survived thresholding for the PBO group at follow-up.

**Suppl Figure 4a. Correlation between pain-evoked brain activity and pain variability.**

The X axis represents pain variability at baseline (top row) and follow-up (bottom row). The y axis represents extracted fMRI BOLD signal from the pain ROI at baseline.

**
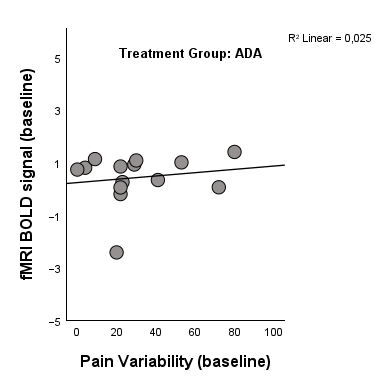

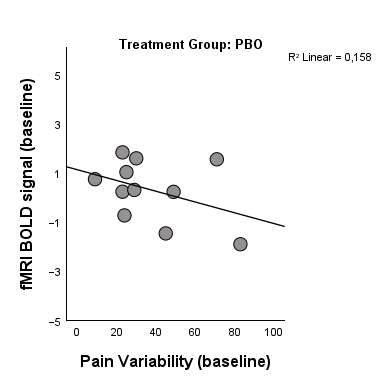
**

**
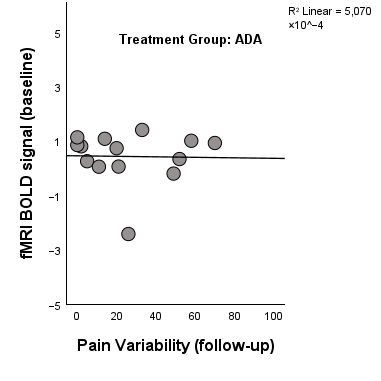

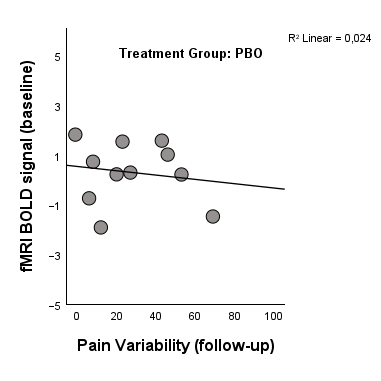
**

**Suppl Figure 4b. Correlation between pain-evoked brain activity and fatigue.**

The X axis represents fatigue at baseline (top row) and follow-up (bottom row). The y axis represents extracted fMRI BOLD signal from the pain ROI at baseline.

**
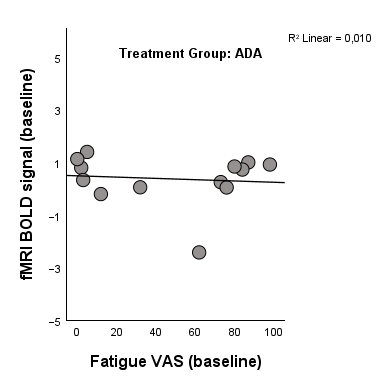

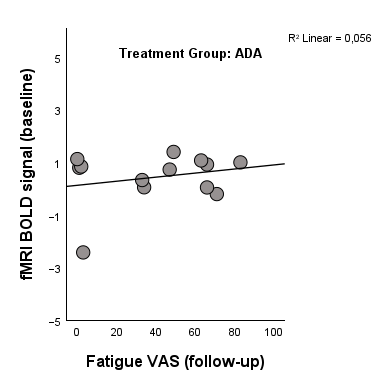

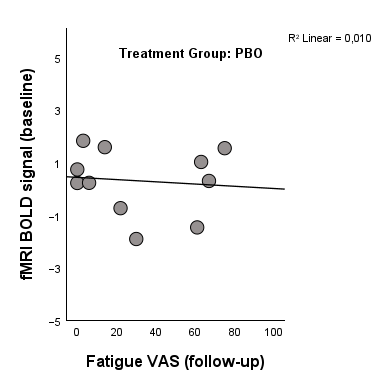

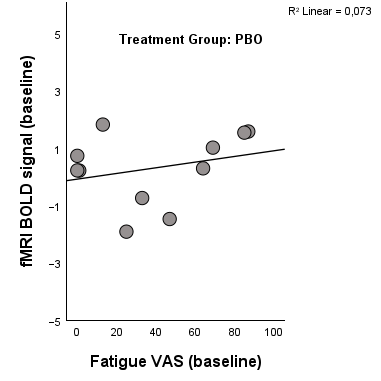
**

**Suppl Figure 4c. Correlation between pain-evoked brain activity and swollen joint count (SJC).**

The X axis represents SJC at baseline (top row) and follow-up (bottom row). The y axis represents extracted fMRI BOLD signal from the pain ROI at baseline.

**
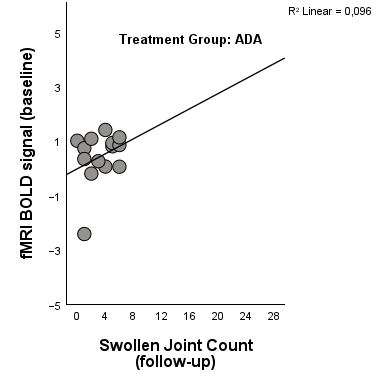

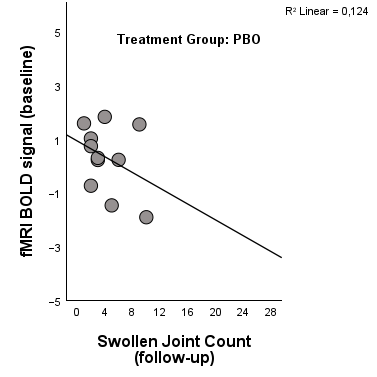

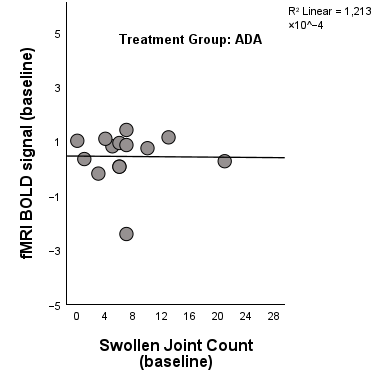

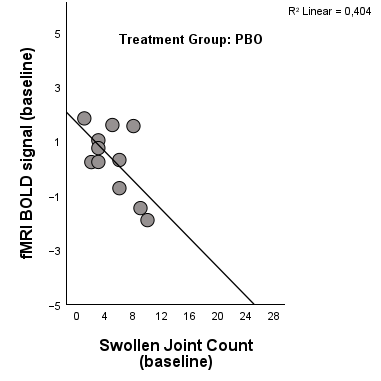
**

**Suppl Figure 4d. Correlation between pain-evoked brain activity and tender joint count (TJC).**

The X axis represents TJC at baseline (top row) and follow-up (bottom row). The y axis represents extracted fMRI BOLD signal from the pain ROI at baseline.


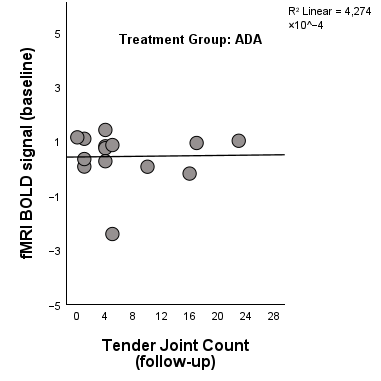

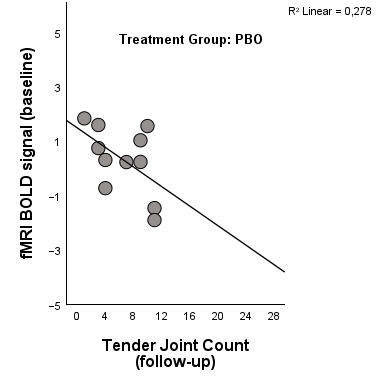

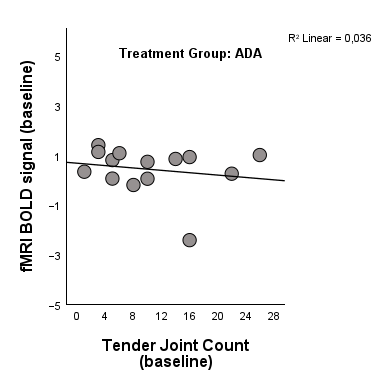

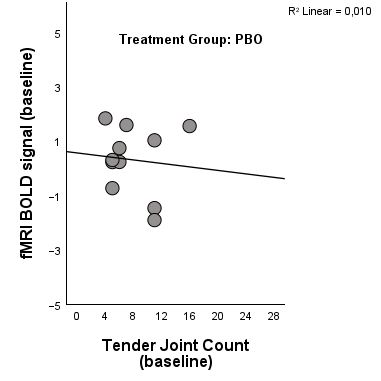

Supplement: Supplementary file 2 — Data S1. Supporting Information. [file ACR2-8-e90069-s002.docx]
